# Associations of RBC counts and incidence of DVT in patients with spinal cord injury: a five year observational retrospective study

**DOI:** 10.1186/s13018-024-04838-1

**Published:** 2024-06-12

**Authors:** Zhang Jinlong, Wang Cheng, He Chengqi

**Affiliations:** 1 Department of Rehabilitation Medicine, West China Hospital, Sichuan University, Chengdu, Sichuan Province 610041 PR China; 2 Department of Rehabilitation Medicine, Division of Life Sciences and Medicine, The First Affiliated Hospital of USTC, University of Science and Technology of China, Hefei, Anhui Province 230031 PR China

**Keywords:** Spinal cord injury, Deep vein thrombosis, Red blood cell counts, D-dimer, Fibrinogen, Anticoagulant therapy

## Abstract

**Background:**

The role of red blood cell (RBC) counts as potential independent risk factors for deep vein thrombosis (DVT) in patients with spinal cord injury (SCI) remains uncertain. This study aims to clarify the associations between RBC counts and DVT incidence among this population.

**Methods:**

A retrospective analysis was performed on 576 patients with SCI admitted to the rehabilitation medicine department from January 1, 2017 to December 31, 2021. After exclusions, 319 patients were analyzed, among which 94 cases of DVT were identified.

**Results:**

Mode of injury, D-dimer and anticoagulant therapy were significant covariates (*P* < 0.05). Age, fibrinogen, D-dimer, anticoagulant therapy and American Spinal Cord Injury Association impairment scale (AIS) grades were associated with RBC counts and DVT incidence (*P* < 0.05). Adjusting for these factors, a 1.00 × 10^12/L increase in RBC counts correlated with a 45% decrease in DVT incidence (*P* = 0.042), revealing a “U” shaped relationship with a pivot at 4.56 × 10^12/L (*P* < 0.05).

**Conclusion:**

RBC counts below 4.56 × 10^12/L serve as a protective factor against DVT, while counts above this threshold pose a risk. These findings could inform the development of DVT prevention strategies for patients with SCI, emphasizing the need for targeted monitoring and management of RBC counts.

**Supplementary Information:**

The online version contains supplementary material available at 10.1186/s13018-024-04838-1.

## Introduction

Historically, red blood cells (RBCs) were considered mere observers in the process of thrombosis [1]. Recent studies, however, have begun to uncover a more complex role. For instance, increased RBC aggregation has been linked to thrombosis in animal models [2], suggesting that RBCs play an active role in the formation of deep vein thrombosis (DVT). Factors such as high blood pressure(HBP) and anemia have also been identified as independent risk factors for DVT [3]. The composition of thrombi in cardiovascular patients and animal models, as observed through advanced imaging techniques, includes RBCs along with other cellular components(fibrinogen, leukocytes and platelets), indicating their significant involvement in thrombosis [4].

The variability in RBC size, as measured by red blood cell distribution width (RDW), has been associated with pulmonary embolism (PE) severity [5], prediction, and prognosis, suggesting a potential link with DVT as well [6]. Routine blood tests, which measure RBC and white blood cell(WBC) counts, hemoglobin levels, and platelet counts, might enhance current risk assessment models for thromboembolic diseases [7]. Studies have found correlations between these hematological parameters and DVT risk formation in various patient populations [8], including those with fibromyalgia and individuals undergoing spinal surgery, highlighting the importance of coagulation function tests in diagnosing thromboembolic conditions [9–11].

Despite these advances, the specific relationship between complete RBC counts and DVT incidence in patients with spinal cord injury (SCI) in rehabilitation department remains understudied. This gap in knowledge prompted our observational study, aimed at exploring this association to inform preventive strategies against lower extremity DVT in the SCI population.

## Materials and methods

### Study design

The study was structured as an observational retrospective analysis.

### Setting and participants

This retrospective study analyzed 576 patients with SCI admitted to the rehabilitation medicine department of The First Affiliated Hospital of USTC, Division of Life Sciences and Medicine, University of Science and Technology of China between January 1, 2017 and December 31, 2021.

### Procedure

**Fig. 1:**
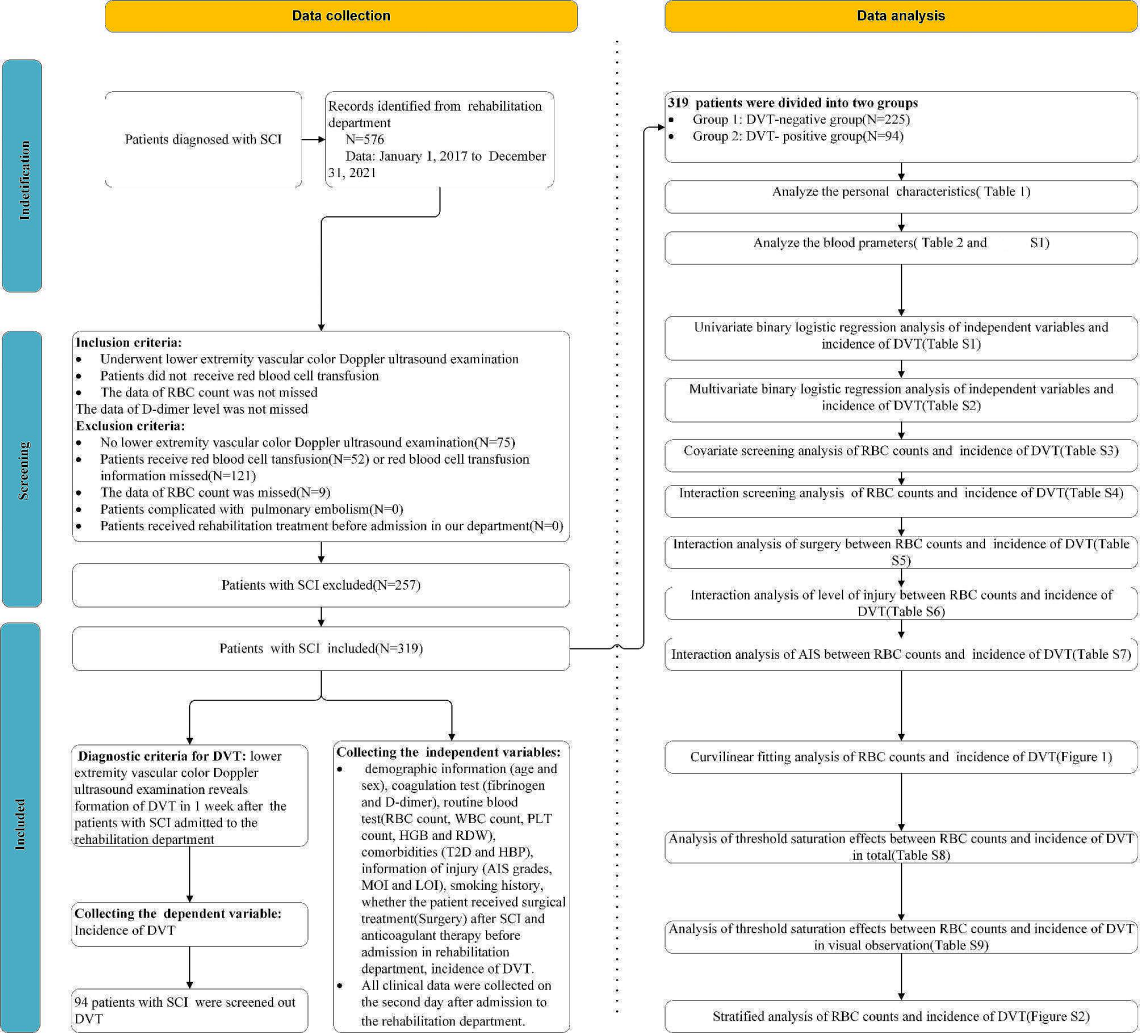
Flowchart of the study Abbreviations: red blood cell count (RBC) count, white blood cell count(WBC) count, red blood cell distribution width(RDW), type 2 diabetes mellitus(T2D), high blood pressure(HBP), American Spinal Cord Injury Association impairment scale(AIS) grades, deep vein thrombosis(DVT)

Following the exclusion of 257 patients due to non-compliance with the inclusion criteria and comply with the exclusion criteria, 319 patients were included in the analysis, of which 94 were identified with DVT via vascular color Doppler ultrasound of the lower extremities. The inclusion and exclusion criteria as presented in figure of flowchart (Fig. 1).

### Data collection

To mitigate selection bias, data collectors were blinded to the study’s objectives. Collected data included demographic information(age and sex), coagulation and routine blood test results(fibrinogen level, D-dimer level, RBC counts, WBC counts, platelet counts, hemoglobin level and RDW), comorbidities(type 2 diabetes mellitus[T2D] and High blood pressure[HBP]), injury details(American Spinal Cord Injury Association impairment scale [AIS] grades, mode of injury, level of injury), smoking history, surgical treatment post-SCI, anticoagulant therapy prior to rehabilitation admission, and DVT incidence (Fig. 1).

### Data analysis

The χ2 or Fisher’s precise test(theoretical frequency < 10)for categorical variables and a student’s *t* test (normal distribution) or Wilcoxon rank sum test with continuity correction(skewed distribution) for continuous variables were used to analyze the differences between the group of DVT(DVT-positive and DVT-negative).

We used imputation way, based on a mean method in the R MI procedure, to account for the missing data of D-dimer and fibrinogen level [12, 13], named variable of D-dimer (imputed) and fibrinogen (imputed), and transformed D-dimer and fibrinogen to a categorical variable named “D-dimer/fibrinogen (indicator)” based on whether the data were missing. Dummy variables were used to indicate the missing covariate values for D-dimer/fibrinogen (indicator), if covariate values were missed; we denoted it as “not recorded” [14] (Tables 1 and Table 2).

**Table 1:** Personal characteristics

DVT	Negative(*n* = 225)	Positive(*n* = 94)	*P* value
	Mean + SD / Median (Min-Max)/N(%)		
Age(years)	48(13–84)	57(18–83)	< 0.001^∗^
Sex			0.492
Female	61 (27.11%)	22 (23.40%)	
Male	164 (72.89%)	72 (76.60%)	
Surgery			0.131
No	10 (4.44%)	1 (1.06%)	
Yes	215 (95.56%)	93 (98.94%)	
AIS grades			0.207
AIS-A	51 (22.67%)	32 (34.04%)	
AIS-B	36 (16.00%)	14 (14.89%)	
AIS-C	59 (26.22%)	24 (25.53%)	
AIS-D	75 (33.33%)	24 (25.53%)	
AIS-E	4 (1.78%)	0 (0.00%)	
Smoking history			0.487
No	174 (77.33%)	76 (80.85%)	
Yes	51 (22.67%)	18 (19.15%)	
Mode of injury			0.185
Traumatic	177 (78.67%)	80 (85.11%)	
Non-traumatic	48 (21.33%)	14 (14.89%)	
T2D			0.879
No	207 (92.00%)	86 (91.49%)	
Yes	18 (8.00%)	8 (8.51%)	
HBP			0.176
No	191 (84.89%)	72 (76.60%)	
Yes	34 (15.11%)	22 (23.40%)	
Level of injury			0.670
Cervical	107 (47.56%)	46 (48.94%)	
Thoracic	64 (28.44%)	31 (32.98%)	
Lumbar	45 (20.00%)	17 (18.09%)	
Cauda equina	4 (1.78%)	0 (0.00%)	
Uncertain	4 (1.78%)	0 (0.00%)	
Medulla oblongata	1 (0.44%)	0 (0.00%)	
Anticoagulant therapy			< 0.001^∗^
No	161(71.56%)	8(8.51%)	
Yes	64(28.44%)	86(91.49%)	

Note: ^∗^*P* < 0.05; DVT: Deep vein thrombosis, AIS: American Spinal Cord Injury Association impairment scale, T2D: type 2 diabetes mellitus, HBP: High blood pressure

**Table 2:** Blood parameters

DVT	Negative(*n* = 225)	Positive(*n* = 94)	*P* value
	Mean + SD / Median (Min-Max)/N(%)		
Fibrinogen(g/L)	3.81 ± 1.33	4.17 ± 1.35	0.029^∗^
Fibrinogen(g/L)(imputed)	3.79 ± 1.32	4.16 ± 1.35	0.023^∗^
Fibrinogen(indicator)			0.883
Recorded	223 (99.11%)	93 (98.94%)	
Not recorded	2 (0.89%)	1 (1.06%)	
D-dimer(mg/L)	1.24(0.06–26.48)	3.42(0.13–50.98)	< 0.001^∗^
D-dimer(mg/L)(imputed)	1.24(0.06–26.48)	3.24(0.13–50.98)	< 0.001^∗^
D-dimer(indicator)			0.871
Recorded	217 (96.44%)	91 (96.81%)	
Not recorded	8 (3.56%)	3 (3.19%)	
RBC count(×10^12^/L)	4.06 ± 0.58	3.59 ± 0.63	< 0.001^∗^
WBC count(×10^9^/L)	8.03 ± 3.39	8.60 ± 3.65	0.179
Platelet count(×10^9^/L)	220.82 ± 76.22	221.96 ± 78.23	0.904
Hemoglobin (g/L)	118.78 ± 18.03	116.73 ± 18.74	0.362
RDW(%)	13.78 ± 6.99	13.34 ± 1.30	0.550

Note: ^∗^*P* < 0.05. RBC: Red blood cell, WBC: White blood cell, RDW: Red blood cell distribution width.

The univariate binary logistic regression method was used to analyze the possible associations of variables (including RBC counts) and the incidence of DVT (Table S1). The multivariate binary logistic regression method was used to analyze the association of RBC counts and the incidence of DVT with two models (Table S2). Model 1 was the non-adjusted model with no covariates adjusted. We used a multivariate logistic regression analysis to screen out the covariates of RBC counts and the incidence of DVT; the variables were excluded if the variance inflation factor(VIF) was greater than ten [15, 16]. Subsequently, potential confounders were selected if they changed the estimates of incidence of DVT by at least 10% in the final models [17, 18](Table S3). Model 2 was the fully-adjusted model with the covariates (presented in Table S3) and unbalanced probable variables adjusted (presented in Table S1).

We used the generalized additive model (GAM) and smooth curve fitting (penalized spline method) to address the nonlinearity or linear association between the RBC count and the incidence of DVT. When nonlinearities were detected, the fold point was first computed using a recursive algorithm; subsequently, a two-piece binary logistic regression model was constructed on either side of the fold point.

In addition, we performed sensitivity analysis to analyze the robustness of the results (stratified and interaction analysis). For continuous variables, we first converted them to categorical variables according to the third quartile (tertile). Then, we performed a subgroup analysis using a layered (stratified) binary logistic regression model (Figure S2). Subsequently, we first used an interaction screening analysis to screen out the possible effect modifiers for association of RBC counts and incidence of DVT (Table S4). And then, we used interaction analysis to validate the possible effect modifiers or variables cannot be screened out due to sample size of subgroups (Table S5-S7).

All statistical analyses were carried out using R software (http://www.R-project.org, The R Foundation, Version:4.3.1) ,EmpowerStats (http://www.empowerstats.com, X&Y Solutions, Inc, Boston, MA, Version:4.2) and Graphpad prism(https://www.graphpad-prism.cn, Version:9.5.1) considering *P* < 0.05 as statistically significant.

## Results

### Population characteristics

Our analysis revealed significant differences between the DVT-positive and DVT-negative groups. Specifically, the DVT-positive group exhibited higher ages, fibrinogen levels, D-dimer levels, and anticoagulant therapy ratios (all *P* < 0.001), while their RBC counts were notably lower (*P* < 0.001). Conversely, no significant differences were observed in terms of sex, surgery, AIS grades, smoking history, T2D, HBP, level of injury, WBC counts, platelet counts, hemoglobin levels, and RDW between two groups (*P* > 0.05).

After employing imputation techniques to address missing data, the adjusted levels of fibrinogen and D-dimer remained significantly higher in the DVT-positive group (*P* < 0.05). The analysis of fibrinogen (indicator) and D-dimer (indicator) variables revealed no significant differences between groups (*P* = 0.883 and *P* = 0.871, respectively), indicating that the missing data did not skew the association between RBC counts and incidence of DVT (Tables 1 and 2 and Figure S1).

### Univariate logistic binary regression analysis

The univariate logistic regression analysis identified several factors significantly associated with the incidence of DVT. Age, with an odds ratio (OR) of 1.05, fibrinogen (*OR* = 1.22), D-dimer (*OR* = 1.19), and anticoagulant therapy (*OR* = 27.04), were positively correlated with DVT incidence, all showing statistical significance (*P* < 0.001 for age, D-dimer, and anticoagulant therapy; *P* = 0.030 for fibrinogen). Conversely, a higher RBC counts was associated with a reduced DVT incidence (*OR* = 0.27, *P* < 0.001). Regarding AIS grades, patients with grade D had a lower risk compared to those with grade A (*OR* = 0.51, *P* = 0.039). Other variables, including surgery, smoking history, mode of injury, T2D, HBP, level of injury, WBC counts, hemoglobin level and RDW, did not show a significant association with DVT incidence (*P* > 0.05) (Table S1).

### Multivariate binary regression analysis

In the multivariate logistic regression analysis, after testing for multicollinearity, no variables were excluded (VIF < 10). The analysis highlighted mode of injury, D-dimer levels, and anticoagulant therapy as significant covariates in the relationship between RBC counts and DVT incidence(Table S3). In the fully adjusted model (Model II), after adjusting the covariates (mode of injury, D-dimer, and anticoagulant therapy) and unbalanced variables (age, fibrinogen, D-dimer, anticoagulant therapy and AIS grades), the association was attenuated but remained significant; a 1.00 × 10^12/L increase in RBC count resulted in a 45% decrease in DVT incidence (*OR* = 0.55, *P* = 0.042) (Table S2).

### Curvilinear fitting analysis

**Fig. 2:**
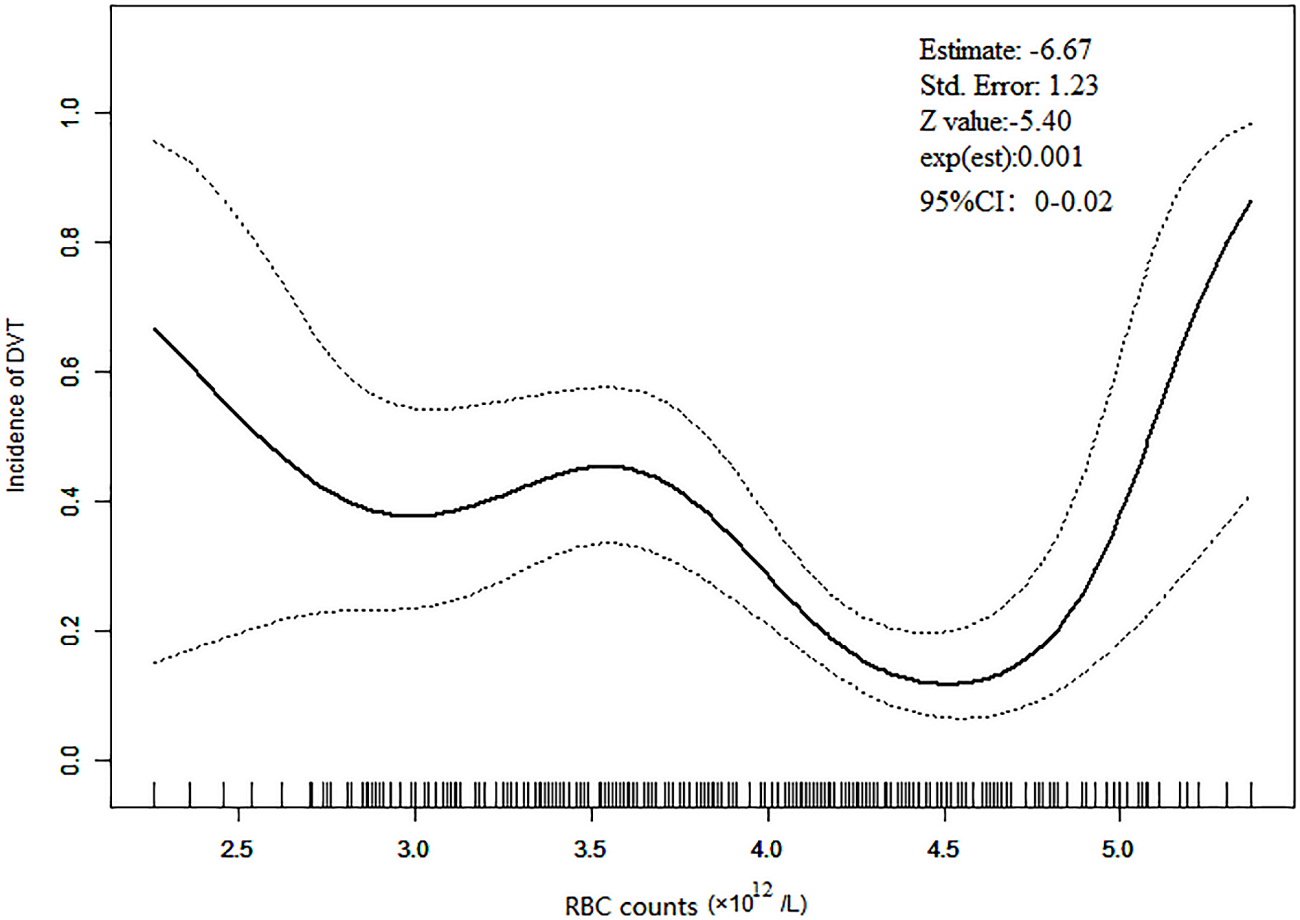
Curvilinear fitting analysis The black continual line is a fitting line of RBC counts and incidence of DVT, and the interval between black dot lines is the 95%CI. Abbreviations: red blood cell count (RBC) count

Upon adjusting for significant covariates and variables found to be unbalanced, the relationship between RBC counts and DVT incidence in patients with SCI was found to resemble a “U” shape. This curvilinear association suggests a complex relationship where both very low and very high RBC counts are associated with increased DVT incidence, with OR of -6.67 and a 95% confidence interval (CI) ranging from 0.00 to 0.02 (Fig. 2).

### Threshold saturation effects analysis

The analysis of the threshold saturation effect between RBC counts and DVT incidence in patients with SCI, after adjusting for significant covariates and unbalanced variables, revealed a fold point at 4.56 × 10^12/L (*P* = 0.010). Below this threshold, a 1.00 × 10^12/L increase in RBC counts was associated with a 65% decrease in DVT incidence (*OR* = 0.35, 95% *CI* = 0.17–0.69, *P* = 0.003). Above the threshold, each 1.00 × 10^12/L increase resulted in a more than 30-fold increase in DVT incidence (*OR* = 31.81, 95% *CI* = 1.67-607.35, *P* = 0.007) (Table S8). According to visual observation of Fig. 2, there appear to be two breakpoints in the relationship between RBC counts and the incidence of DVT, occurring at 3.5 × 10^12/L and 4.5 × 10^12/L (K1 = 3.5, K2 = 4.5). The log-likelihood ratio test found these breakpoints to be statistically significant (*P* < 0.001), indicating a curvilinear association between RBC counts and the incidence of DVT, with breakpoints at 3.5 × 10^12/L and 4.5 × 10^12/L. However, the effect size was not statistically significant when RBC counts were less than 3.5 or more than 4.5 × 10^12/L (*P* = 0.488 and 0.099, respectively). Statistical significance was only observed when RBC counts were between 3.5 and 4.5 × 10^12/L (*OR* = 0.08, *P* < 0.001) (Table S9). Therefore, we believe that the optimal fold point for the relationship between RBC counts and the incidence of DVT was 4.56 × 10^12/L.

### Sensitive analysis

The sensitivity analysis, including stratified and interaction analyses, assessed the robustness of the association between RBC counts and DVT incidence. Stratified analysis indicated a consistent decrease in DVT incidence across various subgroups, with the effect size less than 1 and statistically significant deviation from the reference in most groups (*P* < 0.05). Notably, subgroups based on non-traumatic mode of injury, presence of T2D, and lower tertiles of age and D-dimer levels showed non-significant associations, although the trend of decreased incidence remained(*P* > 0.05) (Figure S1).

Interaction screening did not identify any significant effect modifiers, indicating a broadly applicable relationship between RBC counts and DVT incidence across different patient characteristics (*P* interaction value > 0.05) (Table S4). Despite limitations in subgroup sizes for surgery, level of injury, and AIS grades, subsequent analyses confirmed these variables did not modify the effect of RBC counts on DVT incidence (Table S5-S7).

## Discussion

Mode of injury, D-dimer levels and anticoagulant therapy were identified as significant covariates influencing the relationship between RBC counts and the incidence of DVT in patients with SCI (*P* < 0.05). Age, fibrinogen levels, D-dimer levels, anticoagulant therapy, and AIS grades were found to be critical in this association (*P* < 0.05). Adjusting for these factors revealed that an increase in RBC counts by 1.00 × 10^12/L resulted in a 45% reduction in DVT incidence (*P* < 0.05). Furthermore, the analysis demonstrated a “U” shaped curvilinear relationship between RBC counts and DVT incidence, pinpointing a threshold (fold point) at 4.56 × 10^12/L, beyond which the protective effect of higher RBC count diminishes, and risk increases.

Patients with SCI face a significantly heightened risk of venous thromboembolism (VTE) [19], with mortality rates remaining elevated for six months post-injury, particularly following non-operatively managed traumatic spinal fractures [20]. An epidemiological study revealed 157 cases complicated with DVT (11.71%) for 1341 patients with SCI during the hospitalization period in Northwest China [21]. A meta-analysis identified 9 risk factors (old age, male sex, complete paralysis, and personal/family history of VTE, smoking history, lower limb /pelvic fracture, lack of compression therapy, paraplegia and diabetes) for VTE in patients with SCI [22]. Older patients (≥ 75 years) were easy to experience thromboembolic events [23], and age of ≥ 65 years was found significantly associated with DVT for SCI at rehabilitation unit [24]. Another study found older age (≥ 50 years) and more severe neurological impairment (AIS A, B, and C) were independent risk factors for VTE [22]. The elderly patients (≥ 70 years) with traumatic SCI had a significantly higher VTE incidence and mortality rates than younger patients (< 70 years) [25]. Older age and higher D-dimer levels were also associated with a higher risk of VTE [26, 27]. Our previous research also found age was associated with the incidence of DVT [28]. Besides older age, patients with traumatic injury also had a higher risk of VTE [29]. Interestingly, while certain studies highlight differences in DVT incidence between traumatic and non-traumatic SCI cases [30], others find no significant variation [31]. Therefore, it can be seen that mode of injury, age and AIS grades were closely related to the risk of lower limb DVT in patients with SCI. This study found mode of injury was a covariate, age and AIS grades were unbalanced variable for association of RBC count and the incidence of DVT.

Several patients with SCI were observed to have DVT by Doppler angiography during rehabilitation [32]. Notably, for sub-acute and even chronic patients with SCI had disturbed coagulation and fibrynolitic system, regardless of whether VTE was formed or not [33]. Research indicates D-dimer levels in SCI adults were markedly 70% higher in adults with SCI compared with non-injured adults [34]. Coagulation-related parameters (D-dimer and fibrinogen) of the hospitalized patients with SCI could be used to predict the occurrence of VTE, plasma D-dimer ≥ 0.54 mg/L, plasma fibrinogen ≥ 3.75 g/L were found positively correlated to VTE [35]. D-dimer level was a useful screening parameter [36], and was considered to be the highest diagnostic value among other risk factors(decreased lower extremity muscle strength, time from injury to admission) for DVT in patients with SCI [37]. Moreover, combining D-dimer assessment with ultrasound screening enhances VTE detection in acute SCI patients, surpassing the effectiveness of D-dimer testing alone [38]. Fibrinogen level was also associated with DVT, a higher d-dimer/fibrinogen ratio was found independently associated with a higher DVT risk formation in a dose-dependent manner in patients with cervical SCI [39]. Anticoagulation therapy can decrease the VTE formation risk after SCI [40], or spinal surgery [41]. Rehabilitation treatment could also reduce the DVT risk formation in patients with SCI [42]. The participated in our study did not receive rehabilitation treatment before being enrolled in the rehabilitation medicine department. This study discovered that D-dimer and anticoagulation therapy were significant factors, while fibrinogen levels, alongside D-dimer and anticoagulation therapy, presented as unbalanced variables affecting the RBC counts-DVT incidence relationship.

The relationship between RBC counts and thrombosis has been substantiated by its association with iron concentration in clots among acute ischemic stroke patients [43]. Furthermore, RBCs play a pivotal role in the antithrombotic effect of aspirin in ischemic thrombotic diseases [44], suggesting a link between RBC counts and thrombosis. As DVT is mainly composed of RBCs [45], which may lead to a decrease in the complete RBC counts. In this study, after adjusting for covariates and unbalanced variables, the RBC counts showed a “U” shape curvilinear association with the DVT incidence for patients with SCI, and the fold point was 4.56 × 10^12^/L.

Certainly, our study has a few limitations. Firstly, the observational retrospective nature of the study limits the ability to establish causality between RBC counts and DVT incidence. Longitudinal studies are necessary to confirm these findings and understand the temporal dynamics of RBC counts in relation to DVT incidence. Secondly, due to the complex impact of red blood cell transfusion on VTE, in order to make the results more reliable, we excluded these patients. Thirdly, without longitudinal data, the study cannot assess the long-term effects of RBC counts on DVT incidence or the sustainability of the observed protective and risk thresholds beyond the rehabilitation phases of SCI.

Despite these limitations, the study provides critical insights into the relationship between RBC counts and DVT formation risk in patients with SCI enrolled in rehabilitation department, offering a foundation for future research and clinical practice improvements. Addressing these limitations through longitudinal studies and broader population analyses will be crucial in further elucidating this relationship and developing effective preventive strategies.

## Conclusions

RBC counts below 4.56 × 10^12/L serve as a protective factor against DVT, while counts above this threshold pose a risk. These findings could inform the development of DVT prevention strategies for patients with SCI, emphasizing the need for targeted monitoring and management of RBC counts.

***List of abbreviations***.

Red blood cell (RBC), deep vein thrombosis (DVT), high blood pressure(HBP), red blood cell distribution width (RDW), pulmonary embolism (PE), white blood cell(WBC), spinal cord injury (SCI), type 2 diabetes mellitus(T2D), American Spinal Cord Injury Association impairment scale (AIS), variance inflation factor(VIF), generalized additive model (GAM), odds ratio (OR), venous thromboembolism (VTE).

### Electronic supplementary material

Below is the link to the electronic supplementary material.

Supplementary Material 1

## Data Availability

Data and materials used in this study are available upon reasonable request from the first and corresponding author. Additionally, the original data can be accessed through the following link: https://osf.io/ax27c/?view_only=c1f4ded911174c0f9e62cdf5ee89326a.
