# Supplementary material for: Associations of RBC counts and incidence of DVT in patients with spinal cord injury: a five year observational retrospective study
Source: J Orthop Surg Res. 2024 Jun 12;19:349. doi: 10.1186/s13018-024-04838-1 (PMC11167836; doi:10.1186/s13018-024-04838-1)

**Lists:**

**TableS1:** Univariate binary logistic regression analysis

**Table** **S2:** Multivariate binary logistic regression analysis

**Table S3:** Covariate screening analysis

**Table S4:** Interaction screening analysis

**Table S5:** Interaction analysis of surgery between RBC counts and incidence of DVT

**Table S6:** Interaction analysis of Level of injury between RBC counts and incidence of DVT

**Table S7:** Interaction analysis of AIS grades between RBC counts and incidence of DVT

**Table S8:** Analysis of threshold saturation effects between RBC counts and incidence of DVT in total

**Table S9:** Analysis of threshold saturation effects between RBC counts and incidence of DVT in visual observation

**Figure S1:** Box plot and comparing the RBC counts in both groups

**Figure S2:** Stratified analysis of RBC counts and incidence of DVT

**Abbreviations:** red blood cell count (RBC) count, white blood cell count(WBC) count, red blood cell distribution width(RDW), type 2 diabetes mellitus(T2D), high blood pressure(HBP), American Spinal Cord Injury Association impairment scale(AIS) grades, deep vein thrombosis(DVT)

**Table S1:** Univariate binary logistic regression analysis

|  | Statistics | Incidence of DVT | *P* value |
| --- | --- | --- | --- |
|  | Mean+SD / Median (Min-Max)/N(%) | OR (95%CI) |  |
| Age(years) | 51(13-84) | 1.05 (1.03, 1.07) | <0.001^*^ |
| Sex |  |  |  |
| Female | 83 (26.02%) | Reference |  |
| Male | 236 (73.98%) | 1.22 (0.69, 2.13) | 0.492 |
| Surgery |  |  |  |
| No | 11 (3.45%) | Reference |  |
| Yes | 308 (96.55%) | 4.33 (0.55, 34.27) | 0.165 |
| AIS grades |  |  |  |
| AIS-A | 83 (26.02%) | Reference |  |
| AIS-B | 50 (15.67%) | 0.62 (0.29, 1.32) | 0.217 |
| AIS-C | 83 (26.02%) | 0.65 (0.34, 1.24) | 0.190 |
| AIS-D | 99 (31.03%) | 0.51 (0.27, 0.96) | 0.039^*^ |
| AIS-E | 4 (1.25%) | - | - |
| Smoking history |  |  |  |
| No | 250 (78.37%) | Reference |  |
| Yes | 69 (21.63%) | 0.81 (0.44, 1.47) | 0.487 |
| Mode of injury |  |  |  |
| Traumatic | 257 (80.56%) | Reference |  |
| Non-traumatic | 62 (19.44%) | 0.65 (0.34, 1.24) | 0.187 |
| T2D |  |  |  |
| No | 293 (91.85%) | Reference |  |
| Yes | 26 (8.15%) | 1.07 (0.45, 2.55) | 0.879 |
| HBP |  |  |  |
| No | 263 (82.45%) | Reference |  |
| Yes | 56 (17.55%) | 1.72 (0.94, 3.13) | 0.078 |
| Level of injury |  |  |  |
| Cervical | 153 (47.96%) | Reference |  |
| Thoracic | 95 (29.78%) | 1.13 (0.65, 1.95) | 0.671 |
| Lumbar | 62 (19.44%) | 0.88 (0.46, 1.69) | 0.700 |
| Cauda equina | 4 (1.25%) | - | - |
| Uncertain | 4 (1.25%) | - | - |
| Medulla oblongata | 1 (0.31%) | - | - |
| Anticoagulation therapy |  |  |  |
| No | 169(52.98%) | Reference |  |
| Yes | 150(47.02%) | 27.04(12.39-59.00) | <0.001^*^ |
| Fibrinogen(g/L) | 3.91 ± 1.34 | 1.22 (1.02, 1.46) | 0.030^*^ |
| D-dimer(mg/L) | 1.66(0.06-50.98) | 1.19 (1.11, 1.26) | <0.001^*^ |
| RBC counts(×10^12^/L) | 3.92 ± 0.63 | 0.27 (0.17, 0.42) | <0.001^*^ |
| WBC counts(×10^9^/L) | 8.20 ± 3.47 | 1.05 (0.98, 1.12) | 0.180 |
| Platelet counts(×10^9^/L) | 221.15 ± 76.70 | 1.00 (1.00, 1.00) | 0.903 |
| Hemoglobin (g/L) | 118.18 ± 18.24 | 0.99 (0.98, 1.01) | 0.361 |
| RDW(%) | 13.65 ± 5.91 | 0.98 (0.90, 1.06) | 0.602 |

Note: ^*^*P*<0.05. RBC count: red blood cell count, WBC count: white blood cell count, RDW: red blood cell distribution width, T2D:type 2 diabetes mellitus, HBP: high blood pressure, AIS grades:American Spinal Cord Injury Association impairment scale grades, DVT:deep vein thrombosis

**Table S2:** Multivariate binary logistic regression analysis

**Legend:** Model I: Non-adjusted; Model II: Adjusted for modes of injury, D-dimer, anticoagulation therapy, age, fibrinogen and AIS grades;^*^*P*<0.05. RBC count: red blood cell count

| Exposure | Model I | Model II |
| --- | --- | --- |
|  | OR (95%CI) *P* value | |
| RBC counts(×10^12^/L) | 0.27 (0.17, 0.42) <0.001^*^ | 0.55 (0.31, 0.98) 0.042^*^ |

**Table S3:** Covariate screening analysis

**Legend:**^*^represents a change of >10% with the initial regression coefficient; RBC count: red blood cell count, WBC count: white blood cell count, RDW: red blood cell distribution width, T2D:type 2 diabetes mellitus, HBP: high blood pressure, AIS grades:American Spinal Cord Injury Association impairment scale grades, DVT: deep vein thrombosis

| Variable | Regression coefficient of RBC counts | | Covariate |
| --- | --- | --- | --- |
|  | Basic model | Complete model |  |
| Initial regression coefficient | -1.26 | -0.88 |  |
| Age(years) | -1.16 | -0.97 |  |
| Sex | -1.30 | -0.80 |  |
| Surgery | -1.30 | -0.87 |  |
| AIS grades | -1.21 | -0.84 |  |
| Smoking history | -1.28 | -0.79 |  |
| Mode of injury | -1.34 | -0.73^*^ | Yes |
| T2D | -1.26 | -0.88 |  |
| HBP | -1.29 | -0.89 |  |
| Level of injury | -1.28 | -0.88 |  |
| Anticoagulation therapy | -0.79^*^ | -0.97 | Yes |
| Fibrinogen(g/L) | -1.22 | -0.88 |  |
| D-dimer(mg/L) | -0.96^*^ | -1.02^*^ | Yes |
| WBC counts(×10^9^/L) | -1.25 | -0.84 |  |
| Platelet counts(×10^9^/L) | -1.26 | -0.86 |  |
| Hemoglobin(g/L) | -1.26 | -0.88 |  |
| RDW(%) | -1.27 | -0.89 |  |

**Table S4:** Interaction screening analysis

**Legend:** Surgery, level of injury and AIS cannot be computed; ^*^*P*<0.05; RBC count: red blood cell count, WBC count: white blood cell count, RDW: red blood cell distribution width, T2D:type 2 diabetes mellitus, HBP: high blood pressure, AIS grades: American Spinal Cord Injury Association impairment scale grades, DVT: deep vein thrombosis;

| Y: Incidence of DVT | N | OR | 95%CI Low | 95%CI High | *P* value | *P* interaction value |
| --- | --- | --- | --- | --- | --- | --- |
| Age(years) Tertile |  |  |  |  |  | 0.619 |
| Low | 103 | 0.42 | 0.17 | 1.07 | 0.069 |  |
| Middle | 96 | 0.34 | 0.15 | 0.76 | 0.009^*^ |  |
| High | 120 | 0.24 | 0.12 | 0.49 | <0.001^*^ |  |
| Total | 319 | 0.31 | 0.19 | 0.49 | <0.001^*^ |  |
| Sex |  |  |  |  |  | 0.150 |
| Female | 83 | 0.11 | 0.03 | 0.42 | 0.001^*^ |  |
| Male | 236 | 0.30 | 0.19 | 0.48 | <0.001^*^ |  |
| Total | 319 | 0.26 | 0.17 | 0.41 | <0.001^*^ |  |
| Smoking history |  |  |  |  |  | 0.264 |
| No | 250 | 0.23 | 0.14 | 0.39 | <0.001^*^ |  |
| Yes | 69 | 0.41 | 0.17 | 1.00 | 0.050 |  |
| Total | 319 | 0.26 | 0.17 | 0.41 | <0.001^*^ |  |
| Mode of injury |  |  |  |  |  | 0.479 |
| Traumatic | 257 | 0.25 | 0.15 | 0.42 | <0.001^*^ |  |
| Non-traumatic | 62 | 0.38 | 0.14 | 1.01 | 0.052 |  |
| Total | 319 | 0.27 | 0.18 | 0.43 | <0.001^*^ |  |
| T2D |  |  |  |  |  | 0.344 |
| No | 293 | 0.25 | 0.16 | 0.40 | <0.001^*^ |  |
| Yes | 26 | 0.49 | 0.14 | 1.80 | 0.285 |  |
| Total | 319 | 0.27 | 0.17 | 0.42 | <0.001^*^ |  |
| Anticoagulation therapy |  |  |  |  |  | 0.118 |
| No | 169 | 0.17 | 0.05 | 0.64 | 0.009^*^ |  |
| Yes | 150 | 0.52 | 0.30 | 0.90 | 0.019^*^ |  |
| Total | 319 | 0.44 | 0.26 | 0.72 | 0.001* |  |
| HBP |  |  |  |  |  | 0.275 |
| No | 263 | 0.23 | 0.14 | 0.39 | <0.001^*^ |  |
| Yes | 56 | 0.42 | 0.17 | 1.00 | 0.050 |  |
| Total | 319 | 0.27 | 0.17 | 0.42 | <0.001^*^ |  |
| Fibrinogen(g/L) Tertile |  |  |  |  |  | 0.877 |
| Low | 106 | 0.22 | 0.09 | 0.57 | 0.002^*^ |  |
| Middle | 102 | 0.28 | 0.13 | 0.62 | 0.002^*^ |  |
| High | 111 | 0.30 | 0.15 | 0.61 | 0.001^*^ |  |
| Total | 319 | 0.27 | 0.17 | 0.43 | <0.001^*^ |  |
| D-dimer(mg/L) Tertile |  |  |  |  |  | 0.469 |
| Low | 106 | 0.56 | 0.18 | 1.72 | 0.311 |  |
| Middle | 106 | 0.44 | 0.20 | 0.92 | 0.031^*^ |  |
| High | 107 | 0.26 | 0.12 | 0.56 | 0.001^*^ |  |
| Total | 319 | 0.37 | 0.23 | 0.59 | <0.001^*^ |  |
| WBC counts(×10^9^/L) Tertile |  |  |  |  |  | 0.464 |
| Low | 106 | 0.18 | 0.08 | 0.44 | <0.001^*^ |  |
| Middle | 106 | 0.29 | 0.13 | 0.64 | 0.002^*^ |  |
| High | 107 | 0.36 | 0.18 | 0.72 | 0.004^*^ |  |
| Total | 319 | 0.28 | 0.18 | 0.44 | <0.001^*^ |  |
| Platelet counts(×10^9^/L) Tertile |  |  |  |  |  | 0.288 |
| Low | 105 | 0.19 | 0.08 | 0.46 | <0.001^*^ |  |
| Middle | 105 | 0.21 | 0.09 | 0.47 | <0.001^*^ |  |
| High | 109 | 0.42 | 0.21 | 0.83 | 0.0129^*^ |  |
| Total | 319 | 0.27 | 0.17 | 0.42 | <0.001^*^ |  |
| Hemoglobin(g/L) Tertile |  |  |  |  |  | 0.311 |
| Low | 103 | 0.34 | 0.16 | 0.68 | 0.003^*^ |  |
| Middle | 107 | 0.14 | 0.05 | 0.37 | <0.001^*^ |  |
| High | 109 | 0.31 | 0.15 | 0.64 | 0.002^*^ |  |
| Total | 319 | 0.26 | 0.17 | 0.41 | <0.001^*^ |  |
| RDW(%) tertile |  |  |  |  |  | 0.254 |
| Low | 98 | 0.28 | 0.12 | 0.65 | 0.003^*^ |  |
| Middle | 103 | 0.43 | 0.21 | 0.87 | 0.020^*^ |  |
| High | 111 | 0.17 | 0.08 | 0.39 | <0.001^*^ |  |
| Total | 312 | 0.28 | 0.18 | 0.43 | <0.001^*^ |  |

**Table S5:** Interaction analysis of surgery between RBC counts and incidence of DVT

**Legend:** Model I adjusted for modes of injury, D-dimer, anticoagulation therapy, age, fibrinogen and AIS grades;^*^*P*<0.05. AIS grades: American Spinal Cord Injury Association impairment scale grades; DVT: deep vein thrombosis;

| Model | Surgery: No | Surgery: Yes | *P* interaction value |
| --- | --- | --- | --- |
|  | OR (95%CI) *P* value | |  |
| Crude | 0.14 (0.00, 23.92) 0.453 | 0.26 (0.17, 0.41) <0.001^*^ | 0.798 |
| Model I | 0.89 (0.02, 43.75) 0.954 | 0.52 (0.29, 0.95) 0.034^*^ | 0.804 |

**Table S6:** Interaction analysis of level of injury between RBC counts and incidence of DVT

**Legend:** Model I adjusted for modes of injury, D-dimer, anticoagulation therapy, age, fibrinogen and AIS grades; “-” means cannot be computed;^*^*P*<0.05; AIS grades: American Spinal Cord Injury Association impairment scale grades; DVT: deep vein thrombosis

| Model | Level of injury: Cervical | Level of injury: Thoracic | Level of injury: Lumbar | Level of injury: Cauda equina | Level of injury: Uncertain | Level of injury: Medulla oblongata | *P* interaction |
| --- | --- | --- | --- | --- | --- | --- | --- |
|  | OR (95%CI) *P* value | | | | | |  |
| Crude | 0.34 (0.17, 0.66) 0.001^*^ | 0.17 (0.07, 0.38) <0.001^*^ | 0.32 (0.13, 0.81) 0.016^*^ | - | - | - | 0.742 |
| Model I | 0.43 (0.19, 0.96) 0.039^*^ | 0.48 (0.17, 1.42) 0.187 | 1.05 (0.34, 3.24) 0.938 | - | - | - | 0.807 |

**Table S7:** Interaction analysis of AIS between RBC counts and incidence of DVT

**Legend:** Model I adjusted for modes of injury, D-dimer, anticoagulation therapy, age, fibrinogen and AIS grades; “-” means cannot be computed;^*^*P*<0.05. AIS grades: American Spinal Cord Injury Association impairment scale grades; DVT: deep vein thrombosis

| Model | AIS: AIS-A | AIS: AIS-B | AIS: AIS-C | AIS: AIS-D | AIS: AIS-E | *P* interaction |
| --- | --- | --- | --- | --- | --- | --- |
|  | OR (95%CI) *P* value | | | | |  |
| Crude | 0.27 (0.12, 0.58) <0.001^*^ | 0.27 (0.07, 0.99) 0.048^*^ | 0.27 (0.11, 0.70) 0.007^*^ | 0.34 (0.15, 0.74) 0.006^*^ | - | 0.995 |
| Model I | 0.67 (0.25, 1.80) 0.423 | 0.36 (0.08, 1.53) 0.165 | 0.50 (0.17, 1.44) 0.197 | 0.62 (0.22, 1.77) 0.371 | - | 0.964 |

**Table S8:** Analysis of threshold saturation effects between RBC counts and incidence of DVT in total

**Legend:** Adjusted for modes of injury, D-dimer, anticoagulation, age, fibrinogen and AIS grades. ^*^*P*<0.05. AIS grades: American Spinal Cord Injury Association impairment scale grades, DVT: deep vein thrombosis;

| Outcome: | Incidence of DVT |
| --- | --- |
|  | OR (95%CI) *P* value |
| Model I: Linear regression coefﬁcient |  |
|  | 0.55 (0.31, 0.98) 0.042^*^ |
| Model II: Curvilinear regression coefﬁcient |  |
| Fold point(K) of RBC count | 4.56 |
| < K regression coefﬁcient 1 | 0.35 (0.17, 0.69) 0.003^*^ |
| > K regression coefﬁcient 2 | 31.81 (1.67, 607.35) 0.007^*^ |
| Logarithmic likelihood ratio test | 0.010^*^ |

**Table S9:** Analysis of threshold saturation effects between RBC counts and incidence of DVT in visual observation

**Legend:** Adjusted for modes of injury, D-dimer, anticoagulation, age, fibrinogen and AIS grades. ^*^*P*<0.05. AIS grades: American Spinal Cord Injury Association impairment scale grades, DVT: deep vein thrombosis;

| Outcome: | Incidence of DVT |
| --- | --- |
|  | OR (95%CI) *P* value |
| Model I: Linear regression coefﬁcient |  |
|  | 0.55 (0.31, 0.98) 0.042^*^ |
| Model II: Curvilinear regression coefﬁcient |  |
| Fold point(K1 and K2) of RBC count | K1=3.5, K2=4.5 |
| < K1 regression coefﬁcient 1 | 1.76 (0.36, 8.61) 0.488 |
| K1 to K2 regression coefﬁcient 2 | 0.08 (0.02, 0.36) 0.001^*^ |
| > K2 regression coefﬁcient 3 | 26.75 (0.54, 1333.66) 0.099 |
| Logarithmic likelihood ratio test | <0.001^*^ |

**Figure S1:** Box plot and comparing the RBC counts in both groups

**Legend:** The X lab is the group of DVT, the Y lab is the level of RBC counts. RBC count: red blood cell count, DVT: deep vein thrombosis;


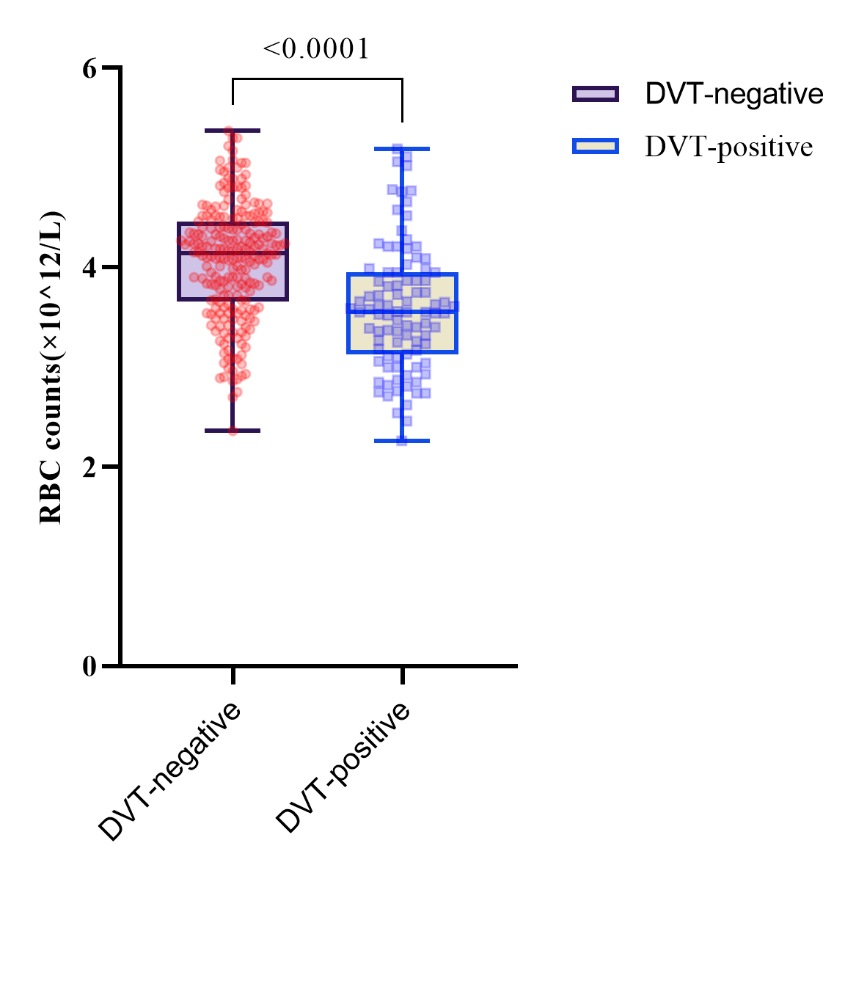


**Figure S1:** Stratified analysis of RBC counts and incidence of DVT

**Legend:** The vertical line is the reference line, the horizontal lines are 95%CI, and the blue squares are plots. “-“means *OR* or *P* value cannot be computed; ^*^*P*<0.05. RBC count: red blood cell count, WBC count: white blood cell count, RDW: red blood cell distribution width, T2D:type 2 diabetes mellitus, HBP: high blood pressure, AIS grades: American Spinal Cord Injury Association impairment scale grades, DVT: deep vein thrombosis;


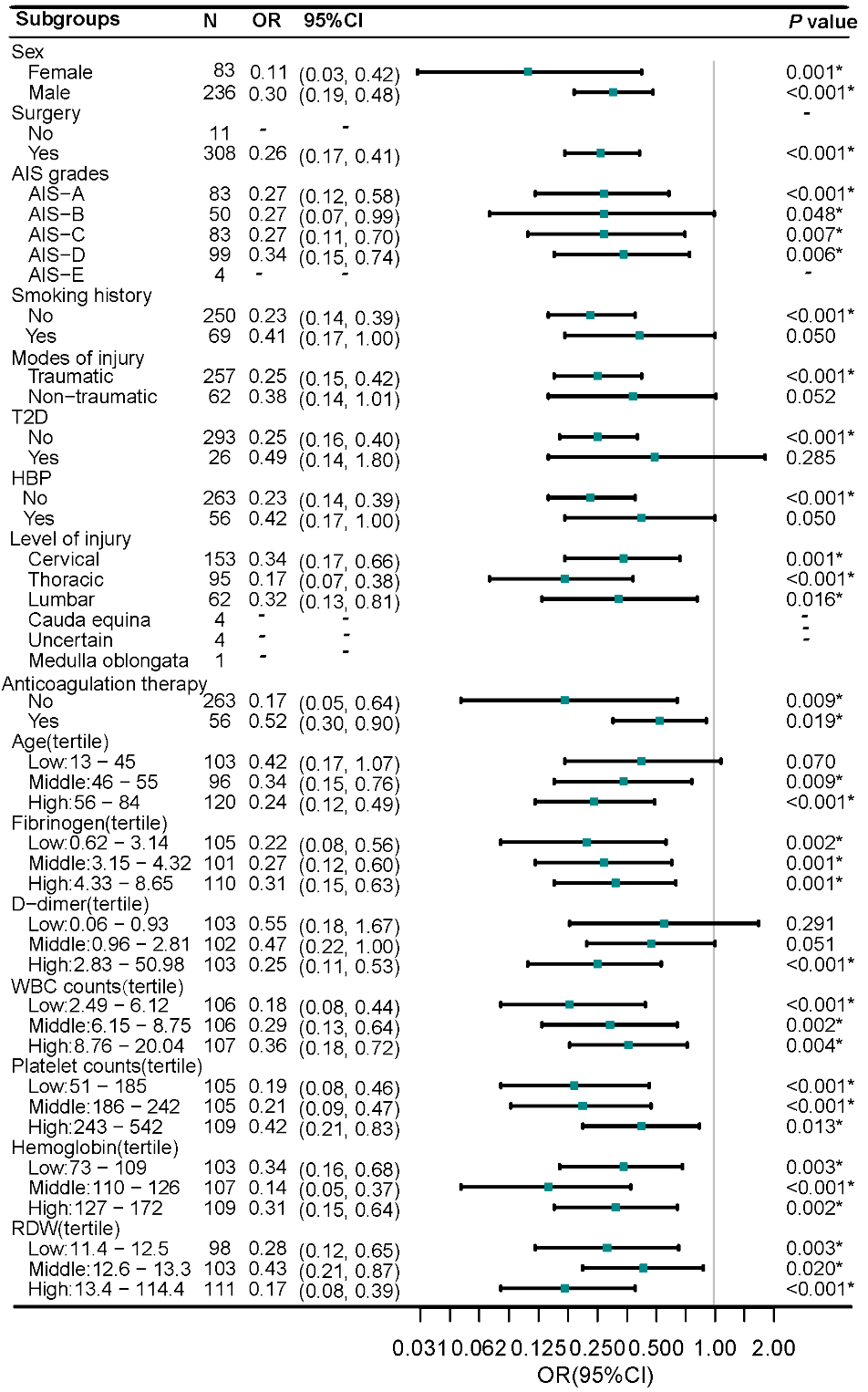

Supplement: Supplementary file 1 — Supplementary Material 1 [file 13018_2024_4838_MOESM1_ESM.docx]
